# Computer-aided system for morphometric mandibular index computation (Using dental panoramic radiographs)

**DOI:** 10.4317/medoral.17637

**Published:** 2012-02-09

**Authors:** Jose López-López, Jose M. Álvarez-López, Enrique Jané-Salas, Albert Estrugo-Devesa, Raul Ayuso-Montero, Eugenio Velasco-Ortega, Juan J. Segura-Egea

**Affiliations:** 1 PhD. MD, DDS. Specialist in Stomatology. School of Dentistry. Department of Odonto-stomatology, University of Barcelona; 2 PhD, DDS. Computer Science research scientist, CBLL, New York University, and Computer Vision Center, Barcelona; 3 MD, DDS. Specialist in Stomatology. School of Dentistry. Department of Odonto-stomatology, University of Barcelona; 4 PhD, DDS. School of Dentistry. Department of Odonto-stomatology, University of Barcelona; 5 PhD, MD, DDS. Specialist in Stomatology. School of Dentistry. Department of Stomatology, University of Sevilla; 6 PhD, MD, DDS. Specialist in Stomatology. School of Dentistry. Department of Endodontics, University of Sevilla

## Abstract

Objective: We propose and validate a computer—aided system to measure three different mandibular indexes: cortical width, panoramic mandibular index and, mandibular alveolar bone resorption index. Study Design: Repeatability and reproducibility of the measurements are analyzed and compared to the manual estimation of the same indexes. Results: The proposed computerized system exhibits superior repeatability and reproducibility rates compared to standard manual methods. Moreover, the time required to perform the measurements using the proposed method is negligible compared to perform the measurements manually. Conclusions: We have proposed a very user friendly computerized method to measure three different morphometric mandibular indexes. From the results we can conclude that the system provides a practical manner to perform these measurements. It does not require an expert examiner and does not take more than 16 seconds per analysis. Thus, it may be suitable to diagnose osteoporosis using dental panoramic radiographs.

** Key words:**Osteoporosis, panoramic mandibular index, cortical width, mandibular alveolar bone resorption index.

## Introduction

Osteoporosis is a systemic bone disease characterized by reduction in bone mass and micro-architectural deterioration of bone tissue. Osteoporosis is the main disease of middle age women, especially menopausal ones. It affects to more than 75 million people in Europe, Japan and the United States. Spain has one of the highest incidences in Europe since there are more than 2.5 millions of affected woman. Further, 40% of these women are not diagnosed yet (1). Although mass bone and calcium metabolism alterations are evident in the pre-menopausal period, the menopause stresses the beginning of bone loss. It is prolonged until life’s end and it is the main cause of bone fractures in older age women (2,3). The Spanish Association Against Osteoporosis (AECOS) estimates an annual hip fracture rate of 60.000 in Spain. Further, the annual rate in the European Union is expected to increase from 414.000 to 972.000 affected people within the next 50 years. Preventive measures and an early diagnose may help decreasing these rates significantly (4). The early onset of osteoporosis can be detected by decreased density of normal bone. The most widely used technique of bone density testing is dual energy X-ray absorptiometry (DXA) (5) consisting of two x-ray beams with differing energy levels aimed at the patient’s bones. Bone density data is interpreted in comparison with what is considered normal for a healthy young person. In this way, the bone density tests calculate the person’s T-score. Then, it is compared against optimal bone density and expressed as number of standard deviations below the average. Finally, according to World Health Organization criteria (6), a T-score greater than 1 is considered normal. A T-score of less than -2.5 is indicative of osteoporosis and a T-score ranging from -1 to -2.5 is considered as a condition of osteopenia (the precursor of osteoporosis).

**Figure 1:**
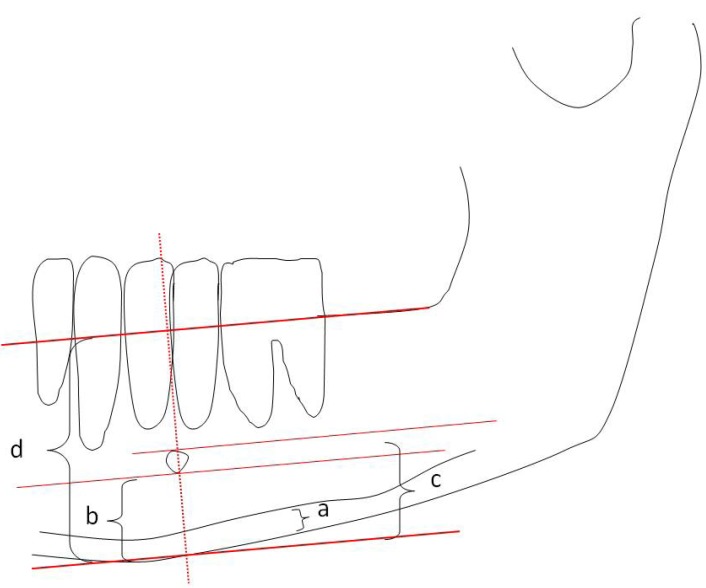
Main measures are defined as: a = Cortical width (CW); a/b= panoramic mandibular index (PMI); d/c= mandibular alveolar bone resorption index (MM ratio).

Recent studies indicate the possibility of assessing the bone mineral density (DMO) analyzing the mandibular bone mineral density (7). In addition, some authors relate osteoporosis to the mandibular residual ridge resorption, periodontal bone loss and tooth loss (8,9). These studies assess and correlate the osteoporosis diagnose with the radiographic results in panoramic radiographs (ortopantomographs, OPG) and periapical radiographs. Further, they highlight the relevance of the dentist in the early onset of this disease. The main dental panoramic radiography measures for the osteoporosis diagnose are: cortical width (CW), also known as mandibular cortical thickness (MTC) (10), panoramic mandibular index (PMI) (11) and, mandibular alveolar bone resorption index (MM ratio) (12); see (Fig. 1). However, the validity of these indexes and related ones such as mandibular angle (13) and the visual morphology of the cortical (14) is under review. Moreover, the precise measurement of these indexes, in terms of repeatability and reproducibility, on dental panoramic radiographs is not practical and is complicated in clinical dental practice (14-16).

These indexes have been widely used in the literature to evaluate the DMO and, thus, to diagnose osteoporosis. Further, considering the orthopantomography is a common practice in dental clinics most authors propose using dental radiography measurements for the early diagnose of osteoporosis. That is, they use these measurements to advise patients to detailed studies of their bone pathology. For instance, Horner K & Devlin H (7) in 1998 conclude that the DMO assessed by DXA is significantly correlated with DMO measurements using MTC and PMI in panoramic radiographs. Further, in 2002, the same authors use measurements of cortical thickness at the dental mandibular foramen (mandibular index, MI), antegonion (antegonial index, AI) and gonion (gonial index, GI) regions to conclude that only MI contributed significantly to a diagnosis of low skeletal DMO (17). White SC et al. (18) in 2005, use MTC and visual cortex analysis described in Klemetti et al. (14) to conclude that both indexes are useful for indentifying subjects having low bone mass. Arifin AZ et al. (19) in 2006, present a computer-aided method to estimate MTC in digital radiographs and to assess the DMO of the patients. Taguchi et al. (20) in 2006, use MTC and the visual cortex analysis combined with a risk factors survey (the osteoporosis self-assessment tool) given to 150 patients to conclude that dentists have sufficient clinical and radiographic information to advise women (younger than 65 years old) to undergo a bone densitometry analysis. In another study, in 2007, Taguchi et al. (21) use MTC and the visual cortex analysis to conclude that postmenopausal women with mandibular distortions usually have low DMO or even osteoporosis. More recently, Vlasiadis et al. (22) in 2008, use mental panoramic radiography indexes as a simple method in the diagnose of osteoporosis. They conclude that there is a relationship between spinal BMD, mandibular cortical width and Klemetti visual cortex analysis.

Therefore, given the difficulty associated to the measurement of mental panoramic radiography indexes we propose a computer-aided system to compute these measures exhibiting, at least, the same efficiency than manual (current) estimation. Hence, the purpose of this study is validating the benefits of estimating the measures using the proposed system in front of the manual method. That is, we aim to prove the usefulness of using a computer-aided system to estimate morphometric measures in orthopantomography.

## Subject and Methods

Ten panoramic radiographs from 10 different subjects are randomly selected from a set of 200 radiographs taken at the University Dental Clinic, University of Barcelona. The subset of radiographs is analyzed by 10 different examiners. They are dentists with more than 3 years of clinical experience. All of them took a 1 hour theory-and-practice course to learn the manual and computer-aided measurement procedures.

Measures to evaluate:

Three different measures are evaluated: cortical width (CW), panoramic mandibular index (PMI) and, mandibular alveolar bone resorption index (MM ratio) (Fig. 1). Manual measurement of these indexes is carried out drawing lines parallel to the long axis of the mandible and tangential to the inferior border of the mandible. Further, a line perpendicular to this tangent intersecting the inferior margin of the mental foramen is drawn. Measurements are taken along this perpendicular line as shown in (Fig. 1). If the mental foramen is visible on both sides of the mandible the measurements are averaged. That is, the mean value of the indexes is considered. Although this is a simple process to estimate the measurements it is not practical providing high inter-observer variations. That is, measurements provided by different dentists are different.

**Figure 2:**
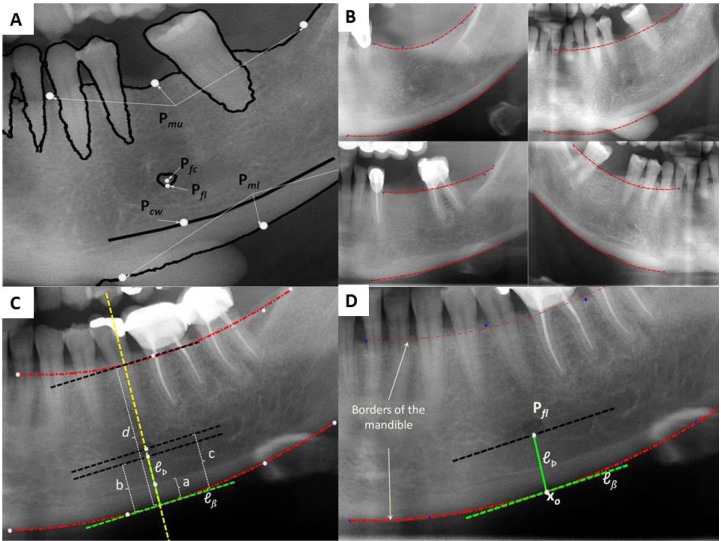
A.The algorithm is based on nine points manually labelled in each image. Pmu: points defining the upper mandibular border. Pml: points defining the lower mandibular border. Pfc: the center of the foramen. Pfl: inferior margin of the foramen. Pcw: points defining the cortical width. 2B. Second order curves (parabolas) are used to model the upper and lower borders of the mandible. These curves can reasonably approximate the real shape of the mandible based on at least three points. 2C. Support lines are drawn to define the slope and position of the mandibular axis. 2D. Different support lines are drawn automatically based on user input. ℓÞ is extended to intersect the upper border of the mandible. All the indices are measured along this extension line. Cortical width is defined by a, Panoramic mandibular index is a/b and the M/M ratio is d/c.

Computer-aided measurement system:

In this section, the proposed algorithm for estimating morphometric mandibular indexes is introduced. The algorithm is based on the knowledge of several points manually annotated in the image (Fig. 2). These points define the upper and lower border of the mandible, the inferior margin and the center of the mental foramen, and the cortical width. Thus, the tedious problem of locating the mandibular axis and measuring distances in a printed orthopantomography is reduced to select nine points in a digital image.

Based on these points the algorithm draws support lines to estimate the morphometric mandibular indexes. In this way, the upper and lower borders are modeled using second order curves (parabolas). Using these parabolic models has two main advantages: its simplicity since only three adjustable coefficients are required, and its physical plausibility since the border of the mandible can be reasonably approximately by a parabolic function in the image plane (Fig. 2). The coefficients of these models are fixed based on input points (Pmu and Pml respectively) in a least square sense. That is, the coefficients of each curve minimize the sum of squared residuals. Residuals are defined as the shortest Euclidian distance between each input point and the resulting curve.

Given the lower border of the mandible and the point defining the inferior margin of the mental foramen Pfl, the support line ℓÞ passing perpendicular to the border and through Pfl is drawn. This line defines the shortest distance between the inferior margin of the foramen and the lower border of the mandible. Then, the slope of the mandibular axis is uniquely defined by the line ℓß tangent to the lower mandibular border at xo, the intersection point between ℓÞ and the lower mandibular border (Fig. 2). Derivatives of the curve are taken and evaluated at xo to derive the slope of ℓß. Then, the slope of ℓÞ is obtained since these two lines are perpendicular. Robustness against inferior foramen margin definition is achieved by selecting multiple points defining the inferior margin of the mental foramen. In this case, the algorithm selects the point corresponding to the minimum distance.

At this point, cortical width (C) is estimated along ℓÞ using Pcw, the cortical width point. This point locates the intersection between the inferior mandibular cortex and ℓÞ. Furthermore, the panoramic mandibular index (PMI) is computed as the ratio of the thickness of the inferior mandibular cortex in the mental region over the distance between the lower border of the mandible and the inferior margin of the mental foramen. This index is also computed along ℓÞ using Pcw.

The remaining is estimating the M/M ratio. This is done extending ℓÞ to intersect the upper border of the mandible as shown in (Fig. 2). Measures are taken along this line between mandible borders and the lower border and Pfc, the center of the mental foramen. If more than one point is selected to define the center of the foramen their centroid is used to compute the measure.

All the measures used to compute the indexes are given in pixels. Nevertheless, the output of the algorithm is given in millimeters using two conversion ratios: distortion ratio and pixel to millimeter ratio. The former refers to the distortion introduced in the imaging process. The latter refers to the distance of the mandible covered by each pixel in the image. These ratios are device dependent. The distortion ratio must be introduced manually whereas the pixel to millimeter ratio can be estimated by comparing manually measured distances in a printed orthopantomography against the number of pixels in the corresponding image.

Finally, the algorithm for computing the morphometric mandibular indexes is summarized as follows: 1) Perform algorithm calibration if required. 2) Load and display the region of interest of an orthopantomography. 3) Select at least three points defining the upper border of the mandible (Pmu). 4) Select at least three points defining the lower border of the mandible (Pml). 5) Select at least one point defining the inferior margin of the mental foramen (Pfl). 6) Select at least one point defining the center of the mental foramen (Pfc). 7) Select the point (Pcw) along the line ℓÞ to define the mandibular inferior cortical width. This line is tangent to the lower mandible border and passes through the inferior foramen margin. 8) Save the results if required.

Methodology:

Three different measurements sets are carried out by each examiner in each orthopantomography with ten minutes break between measurements. OPG are randomly selected at each measurement round. This process is repeated using the manual and computer-aided measurement methods. Further, half examiners perform the manual method first and then, the computerized one. Half examiners perform the computerized method first and then the manual one. Hence, the measurement process for each OPG is summarized as follows:

1) Manual method: 30 measurements per orthopantomography. Each examiner measures 3 times each OPG following the manual method as described in Benson BW et al. (11) and Klemetti E & Kolmakow S (14), (Fig. 1).

2) Computer-aided method: 30 measurements per orthopantomography. Each examiner measures 3 times each OPG using the proposed computer-aided system.

Measurements obtained using the manual procedure are written by the examiner at the end of the three rounds and subsequently an external user introduces these values into a database. Measurements obtained using the computerized method are stored directly into the database. The output of the system is an excel file for each OPG exhibiting the different measurements taken by each examiner.

Statistical Analysis:

Repeatability and reproducibility are used to analyze the results in terms of inter- and intra-examiner agreement respectively. Repeatability refers to the variability of the measurements obtained by one examiner (observer) while measuring the same data repeatedly. Reproducibility is the variability of the measurement system caused by differences in the observer behavior. These measures are estimated through the precision of the measurements expressed as the variance of multiple measurements by a single person. Furthermore, the range of reliability of the measurements is analyzed using the 95% confidence interval. In addition, average time required to estimate the indexes using both methods (manual and computer-aided) is considered. Finally, pair-wise comparisons between manual and computer aided measurements are computed by Wilcoxon significance test (23).

## Results

**Figure 3:**
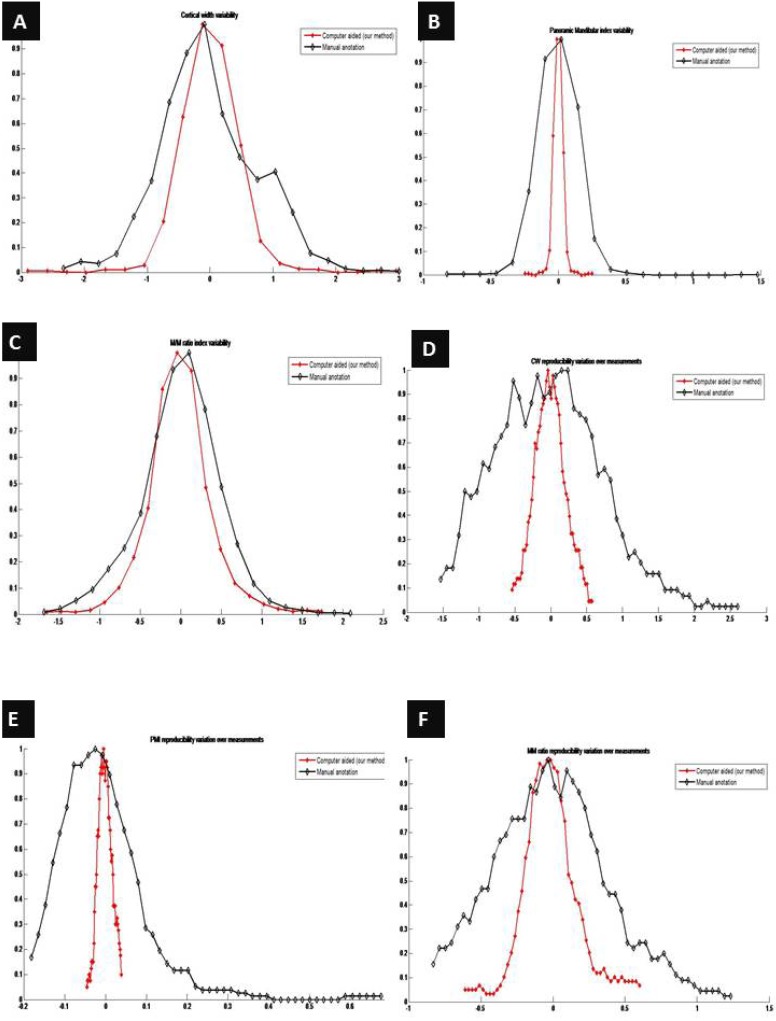
A. Per user cortical width variability. 3B. Per user panoramic index variability. 3C. Per user M/M ratio variability. 3D. Distribution of errors when different examiners measure the same orthopantomography (OPG). 3E. Distribution of errors when different examiners measure the same OPG. 3F. Distribution of errors when different examiners measure the same OPG.

Repeatability of the measures is analyzed measuring the agreement achieved when one user measures the same in different orthopantomographs. ( Table 1) lists the differences per user for each index. Furthermore, the distributions of errors (differences) for all the users are shown in (Fig. 3), for the three indexes respectively. As shown, the variability using the proposed system is lower than manually computing each index. That is, differences between the same user measurements are lower when the examiner takes the benefit of the computer aided system.

**Table 1:**
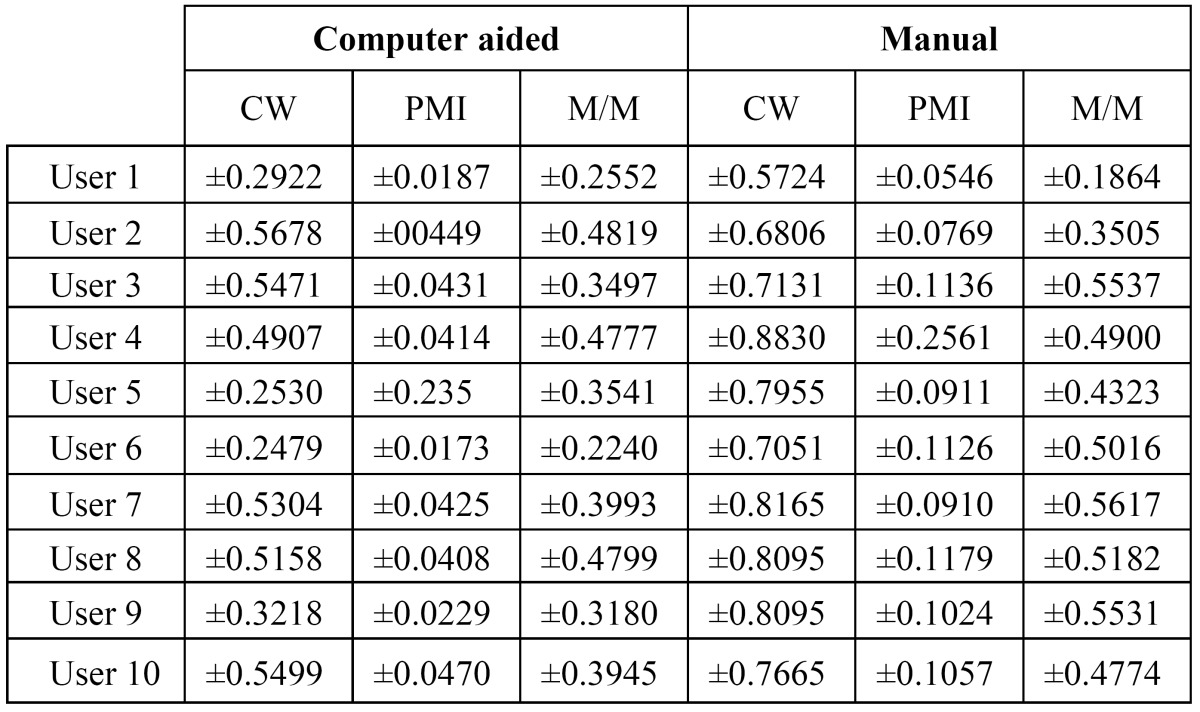
User variability.

Reproducibility of the measurements is analyzed using the average measurements per user. Given an orthopantomography, the average value per user is estimated using the differences between each user measurement over the average measurement from all the users. Each user measurement is computed as the average value of all trials. As a result, the summary of values listed in ( Table 1,  Table 2,  Table 3

**Table 2:**
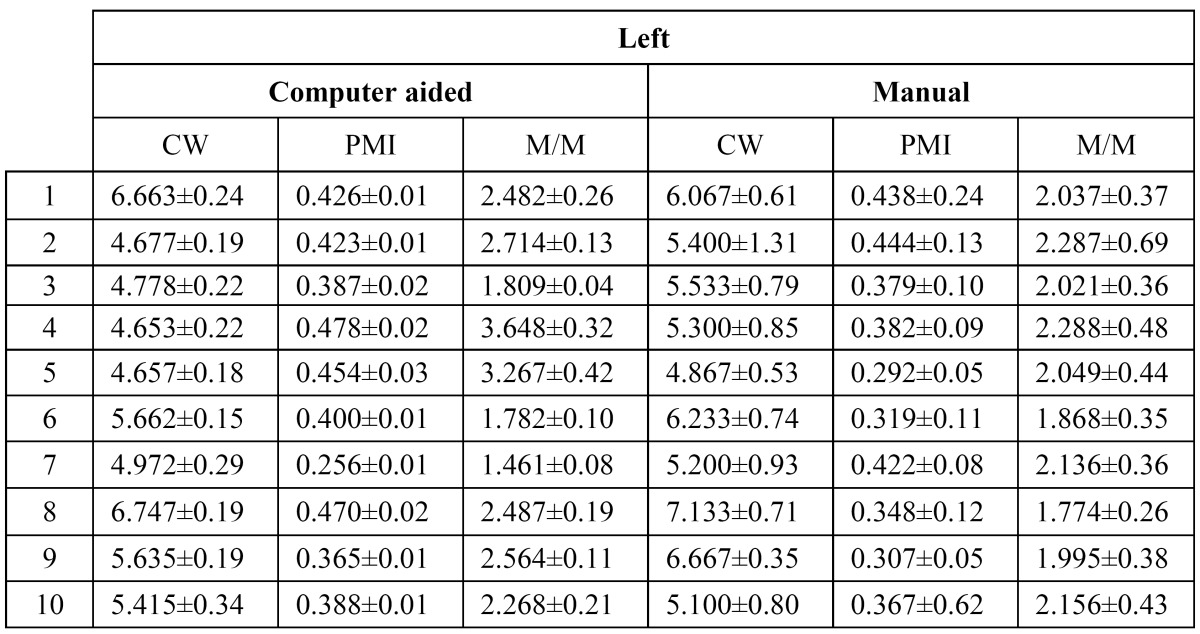
User measurement variability for the left side of each orthopantomography.

**Table 3:**
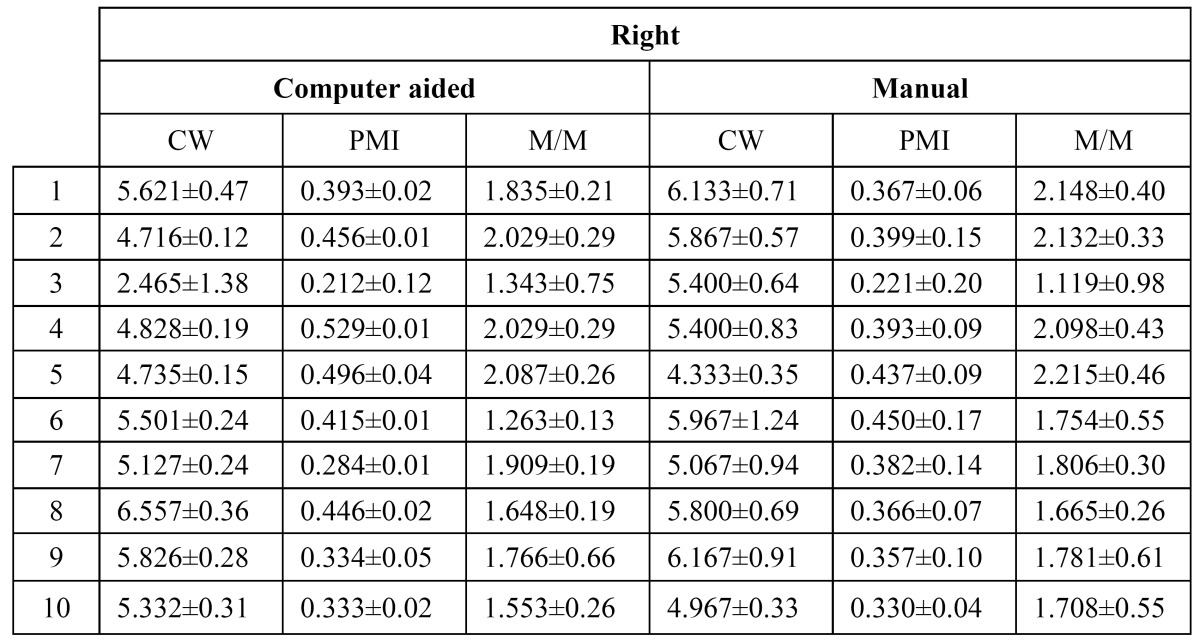
User measurement variability for the right side of each orthopantomography.

Furthermore, three error distributions are obtained, one for each index (Fig. 3). The summary of statistics over these distributions and the Wilcoxon significance test are listed in ( Table 4). From these results, we can conclude that our computer-aided system outperforms the manual procedure in terms of reproducibility (23). Time required for computing the indexes is analyzed. Average time per analysis is listed in ( Table 5).

**Table 4:**
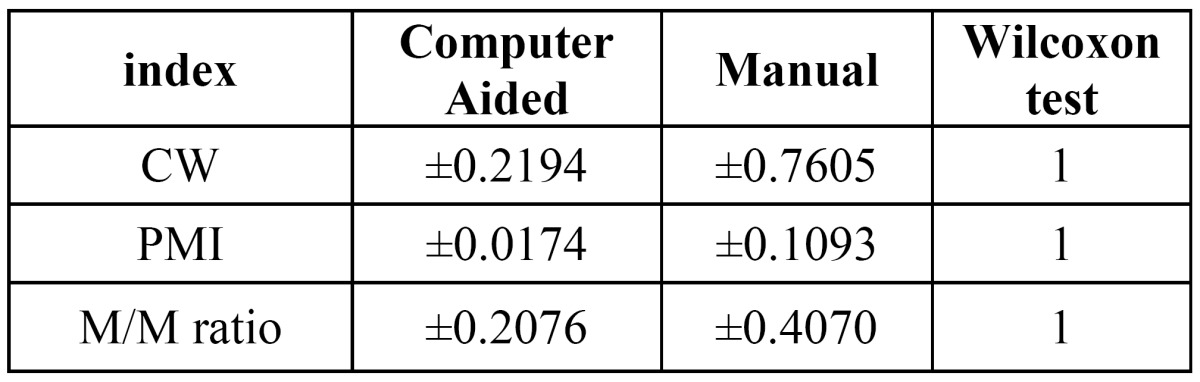
Standard deviation of errors when different examiners measure the same orthopantomography. The third column lists the Wilcoxon significance test for the reproducibility experiment. A positive value (1) indicates that the computer aided system outperforms the manual process.

**Table 5:**
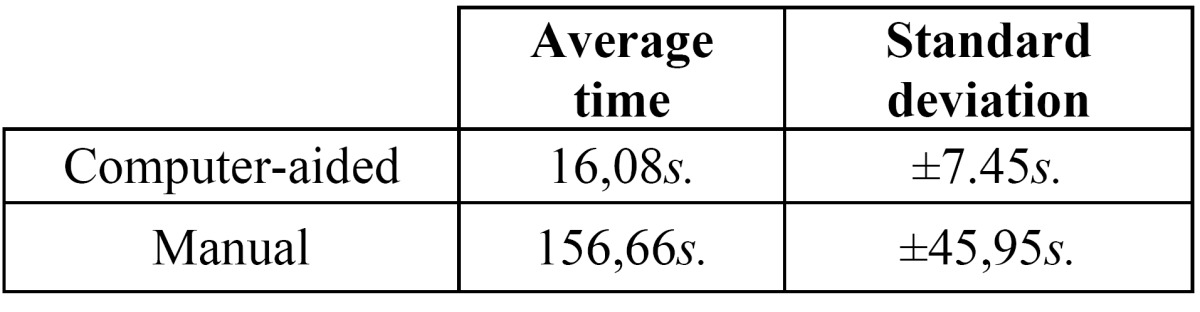
Average time required to measure all three indexes in a single orthopantomography using the manual and the proposed computer-aided methods.

As shown, the average time per orthopantomography using the computer aided system is 16 seconds. However, the average time required to perform the same analysis manually is higher than 2 minutes. Hence, we can conclude than using the computer aided method reduces significantly the required time per analysis. Therefore, the computer-aided method also reduces the user fatigue and, consequently, improves the long term accuracy of the analysis.

## Discussion

There are a number of works using photodensitometric analysis to diagnose osteoporosis. For instance, Nakamoto T et al. (24) in 2008, perform gray level analysis in OPG to identify patients with osteoporosis. Jonasson G et al. (25) in 2001, predict the skeletal DMO using a photodensitometric measurement of the bone mass of the mandible, the trabecular pattern of the alveolar bone and its interdental thickness. Other authors define a criterion to classify trabecular patterns as dense, heterogeneous or sparse (26,27). They conclude that the visual assessment of the trabecular pattern in OPG can be used to identify patients having osteopenia or osteoporosis.

Regarding manual measurements in dental panoramic radiographs, there are only a few works addressing them using a computerized system. Arifin AZ et al. (19) in 2006, use a computer-aided method in digital radiographs to estimate MTC. The system relies on image enhancement techniques to measure MTC. However, since the margins of the cortex are not always clearly defined the system requires manual assistance to perform its task. Further, the variability of the measurements is highly influenced by the expertise of examiners. These authors compare manual and computerized methods to estimate the cortical width. As a result, they conclude that both systems are highly correlated although they do not specify the system used. However, results regarding time or degree of difficulty are not provided (19).

The proposed computer-aided system is user friendly and does not require two much time to estimate the measures. Further, given an ortopantomograph, it is highly repetitive for a given examiner. In addition, given the low examiner fatigue produced, intraexaminer calibration using the repeatability index proposed by Landis JR & Koch GG (28) is feasible. Furthermore, there are not errors associated with the annotation process since it is done automatically. That is, the system provides an excel file as output with all the measurements per ortopantomograph. Finally, the precision of the computer-aided method in the measurements in front of the manual one must be highlighted. Example measures for a single orthopantomography are listed in ( Table 6). As shown, measuring the cortical width manually leads to rough measures. However, using the computer–aided system provides higher accuracy reducing the propagation of errors. On the contrary, there are different difficulties associated to the manual measurement method such as specific illumination, magnification, examiner calibration as proposed in Ledgertod et al. (29) or systematic measurements as proposed by Karayianni et al. (30). Further, the manual procedure is vulnerable to errors when annotating registered measures.

**Table 6:**
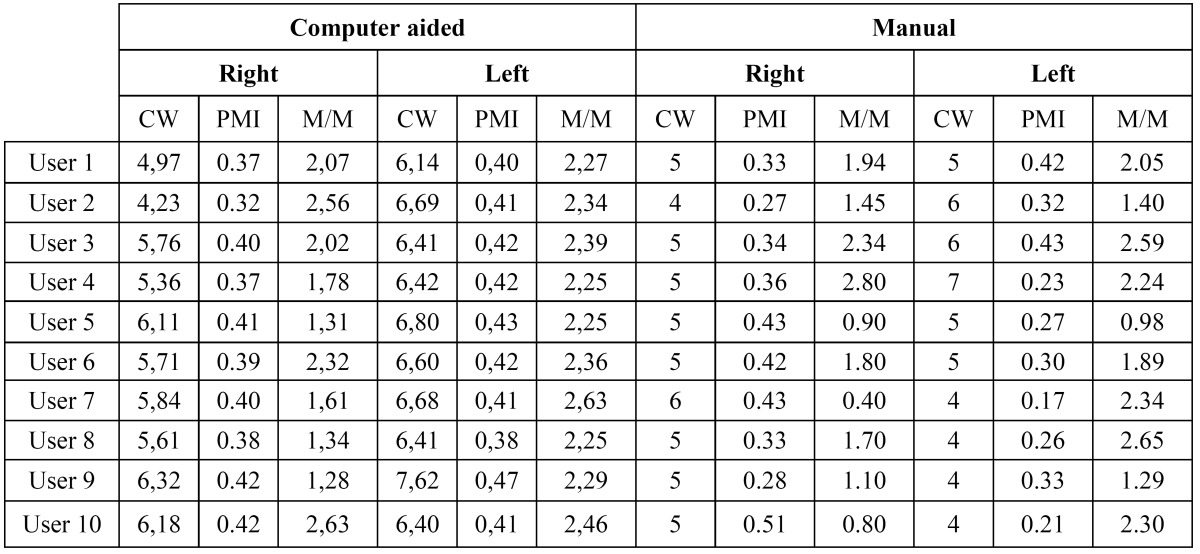
Example measures for a given orthopantomography using both methods: computer-aided and manual.

## Conclusions

We have proposed a very user friendly computerized method to measure three different morphometric mandibular indexes: cortical width, panoramic mandibular index and mandibular alveolar bone resorption index. The proposed method exhibits superior repeatability and reproducibility rates compared to standard manual methods. Moreover, the time required to perform the measurements using the proposed method is negligible compared to perform the measurements manually. From the results we can conclude that the system provides a practical manner to perform these measurements. It does not require an expert examiner and does not take more than 16 seconds per analysis. Thus, it may be suitable to diagnose osteoporosis using dental panoramic radiographs.
